# Antibiotic Conjugated Fluorescent Carbon Dots as a Theranostic Agent for Controlled Drug Release, Bioimaging, and Enhanced Antimicrobial Activity

**DOI:** 10.1155/2014/282193

**Published:** 2014-03-18

**Authors:** Mukeshchand Thakur, Sunil Pandey, Ashmi Mewada, Vaibhav Patil, Monika Khade, Ekta Goshi, Madhuri Sharon

**Affiliations:** N.S.N. Research Center for Nanotechnology and Bionanotechnology, Ambernath, Maharashtra 421505, India

## Abstract

A novel report on microwave assisted synthesis of bright carbon dots (C-dots) using gum arabic (GA) and its use as molecular vehicle to ferry ciprofloxacin hydrochloride, a broad spectrum antibiotic, is reported in the present work. Density gradient centrifugation (DGC) was used to separate different types of C-dots. After careful analysis of the fractions obtained after centrifugation, ciprofloxacin was attached to synthesize ciprofloxacin conjugated with C-dots (Cipro**@**C-dots conjugate). Release of ciprofloxacin was found to be extremely regulated under physiological conditions. Cipro**@**C-dots were found to be biocompatible on Vero cells as compared to free ciprofloxacin (1.2 mM) even at very high concentrations. Bare C-dots (**∼**13 mg mL^−1^) were used for microbial imaging of the simplest eukaryotic model—*Saccharomyces cerevisiae* (yeast). Bright green fluorescent was obtained when live imaging was performed to view yeast cells under fluorescent microscope suggesting C-dots incorporation inside the cells. Cipro**@**C-dots conjugate also showed enhanced antimicrobial activity against both model gram positive and gram negative microorganisms. Thus, the Cipro**@**C-dots conjugate paves not only a way for bioimaging but also an efficient new nanocarrier for controlled drug release with high antimicrobial activity, thereby serving potential tool for theranostics.

## 1. Introduction

Carbon quantum dots or carbon dots (C-dots) have become a colossal designation in the field of material science, since its serendipitous inception in 2004 during separation of multiwalled carbon nanotubes under electrical influence [1]. In medicine and theranostics, C-dots have emerged as new advancement owing to their exceptional biocompatibility [2], typical optical properties [3], nontoxic precursors as carbon sources, high aqueous solubility, and easy surface functionalization, unlike semiconductor quantum dots such as CdTe and CdSe. [4, 5]. Another catchy attribute of C-dots is their photoluminescence (PL) in near-infrared region (NIR) which can be potentially used for photothermal therapy of tumors [6, 7]. There is significant advancement in synthetic protocols for fabrication of fluorescent C-dots over the past few years. Most celebrated among them is microwave mediated synthesis [1], laser ablation of graphite [8], thermal cracking of organic compounds [9], electrooxidation of graphite [10], and oxidation of candle soot [11]. Moreover, there are very few reports on fabrication of C-dots using natural plant materials as carbon source. Recently, C-dot was synthesized using orange juice [12], jaggery, bread, and sugar [13]. These C-dots being made from natural materials become exceptionally biocompatible and cost effective for bulk production.

Due to exceptional biocompatibility C-dots are exploited as versatile drug delivery vehicles for chemotherapeutic payloads [14–17]. Antibiotic conjugation strategy is particularly important for controlled releases of antibiotics since there is increasing microbial resistance due to overdosage of antibiotics [18, 19]. Moreover, we have synthesized C-dots using edible source, making it more biocompatible. We observed sustained release of ciprofloxacin over 24 h making Cipro@C-dots ideal sinks to control pathogenic infections.

## 2. Experimental

### 2.1. Materials and Methods

GA was procured from the local market after ensuring high purity. All the chemicals under experimental considerations were of analytical grade and were used as received.

### 2.2. Characterization

Spectral properties of the C-dots were studied by UV-Vis Spectroscopy (Lambda-25, Perkin Elmer, USA) where the spectrum was recorded at a 1000-fold dilution of the sample. Fluorescence Spectroscopy (Perkin Elmer, USA) was carried in a standard quartz cuvette. 350, 400, 450, and 500 nm were selected as excitation wavelengths. Fourier transform infrared spectroscopy (Brucker) studies were performed within the spectral window 500 to 4000 cm^−1^. HRTEM (Carl Ziess, GmbH, Germany) studies were performed onto a carbon-coated formwar. Crystallinity of C-dots was studied using X-ray diffraction (Phillips, The Nederland). For analysis, samples were dried on glass coverslip. Raman spectra were recorded using Jobin-Yvon Labram spectrometer. Samples were excited using lasers (632.8, 532, and 488 nm) with a spectral resolution of <1.5 cm^−1^. All the spectra were initially baseline corrected with 3rd order polynomial and normalized to the max of the peak intensity. ^1^H NMR analysis was done using Bruker DPX 300 MHz Spectrometer using DMSO-d6 as solvent.

### 2.3. Synthesis and Separation of C-Dots

1 g of GA was dissolved in 10 mL of cold distilled water to obtain light yellow colored solution. To this mixture, 3 mL absolute ethanol (EtOH, 99.99%) and sodium hydroxide (NaOH, 1 M) mixture (in equal volume) were added and subjected to microwave assisted pyrolysis for 5 min till color of the mixture turned to wine red. This mixture was separated by sucrose density gradient centrifugation (SDGC) using 50–100% gradient concentration of sucrose. Three distinct bands were removed carefully and their properties were studied. Bands are referred to B1, B2, and B3 for further discussions. Each fraction was subjected to repeated centrifugation steps to get rid of residual sucrose and pure C-dots were collected by spinning at 8385 ×g for 15 min. On vacuum heating for 8 h, powdered form of black colored C-dots was obtained which was then used to make 100 mg/mL stock solution and stored at −20°C.

### 2.4. Synthesis of Cipro@C-Dot Conjugate

For the synthesis of the above conjugate, 0.5 mL (1000 *μ*M) ciprofloxacin solution was added to 9.5 mL (95 mg/mL) C-dots and stirred for 3 h at 30 ×g. Change in the optical properties of Cipro@C-dots conjugate was studied using UV-Vis Spectroscopy in the spectra window of 200–600 nm with respect to pure C-dots. Further attachments of C-dots and ciprofloxacin were confirmed using Fourier transform infrared (FTIR) and thermogravimetric analysis (TGA). Drug loading efficiency (DLE) of C-dots was calculated using the following equation (see Supplementary Material, Scheme 1a, available online at http://dx.doi.org/10.1155/2014/282193):

(1) DLE=Theoretical  amount  of  drug  loaded−Free  drugTheoretical  amount  of  drug  loaded×100.

### 2.5. Antibiotic Release Studies

2 mL of Cipro@C-dots conjugate was transferred to a fresh dialysis bag (MW cutoff 12–14 kD, Pore size 2.4 nm) and dialyzed against 1% phosphate buffer saline (PBS, pH 7.2) at 37°C. The antibiotic release at regular time intervals (0–48 h) was measured spectrophotometrically at 277 nm. Each time the reading appropriate volume of fresh phosphate buffer saline (PBS, pH 7.2) prewarmed and maintained at 37°C in an incubator was added to the dialysis chamber.

### 2.6. Cytotoxicity Studies

Cytotoxic effect of the Cipro@C-dots conjugate was studied on most commonly used Vero cells using 3-(4,5-dimethyl-2-thiazolyl)-2,5-diphenyltetrazolium bromide (MTT) assay. Vero cells were seeded (3 × 10^5^/mL) in 96 well plates and incubated at 37°C under 5% CO_2_ for 24 h. After satisfactory growth of the cells, growth medium was replaced with the respective test solutions and incubated for 48 h. Finally, C-dots or Cipro@C-dots solution was replaced with MTT (150 *μ*g/mL). Cells were incubated for 2 h at 28 ± 2°C to initiate formation of formazan. After completion of the reaction, medium was replaced with 300 *μ*L of DMSO (Sigma, USA). This conjugate was agitated moderately to dissolve formazan crystals. Finally, the dissolved formazan in DMSO was transferred to fresh 96 well plates and read on microplate reader (Thermo, USA) at 570 nm.

### 2.7. Antimicrobial Studies

For antimicrobial activity studies, the microorganisms were procured from Microbiology Department of N.S.N. Research Center, Ambernath, India. Two representative gram positive* Bacillus subtilis* and* Staphylococcus aureus* and two representative gram negative* Escherichia coli* and* Pseudomonas aeruginosa* were procured. The microbial strains were maintained onto agar slants at 4°C. Mueller-Hinton agar plates were then prepared and spread plated with bacteria. Well-diffusion method was employed for carrying out the antimicrobial activity. Four wells were bored with sterile cork borer. Wells were labeled, respectively, for distilled water as negative control, ciprofloxacin (1 mM) as positive control, and other two equivalent concentrations of test samples, C-dots and Cipro@C-dots conjugate, in each keeping C-dots concentration constant in both the samples.

## 3. Results and Discussion

**Figure 1:**
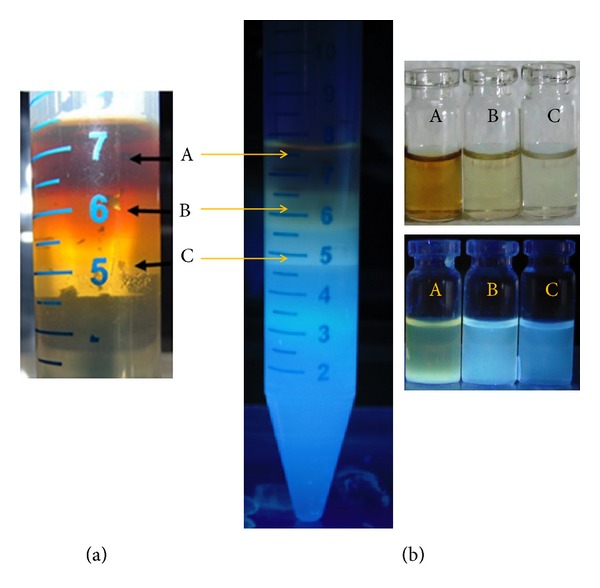
Separation of C-dots using SDGC. (a) Separated bands under ambient light and (b) 250 nm excitation UV lamp. Upper and lower panels show color of the fractions under normal light and UV light, respectively.

GA is extremely branched arabinogalactan polysaccharide [19]. Due to the very high content of branched carbon and proteins, it could act as versatile raw material for the synthesis of highly fluorescent C-dots by microwave assisted carbonization. Color of GA (pale yellow) got transformed to wine red after heating for 5 min under the influence of EtOH and NaOH as surface passivation agents. This was color marker for synthesis of C-dots as per previous studies [20]. Under UV light (*λ* = 365 nm), turbid green fluorescence was observed, which may be due to presence of partially burnt carbonaceous materials along with graphene oxide (GO). Nanoparticulate systems never possess monodispersed particles by virtue of strange quantum mechanical attributes and thermodynamics at nanoscale [21]. Therefore, for efficient application of C-dots, its separation became mandatory using SDGC. SDGC separates nanoparticles based on their hydrodynamic properties. Due to negligible impact of gravity, inertia, and dominant thermal energies, separation of ultrasmall particles such as C-dots is not possible by simple centrifugation techniques [22]. Fractions are separated based on their densities with respect to sucrose gradient. Three discrete bands were seen with different fluorescence intensities as shown in Figure 1. For systematic discussions on optical as well as morphological properties of isolated bands, B1, B2, and B3 are considered separately (SI S1 for quantum yield values).

**Figure 2:**
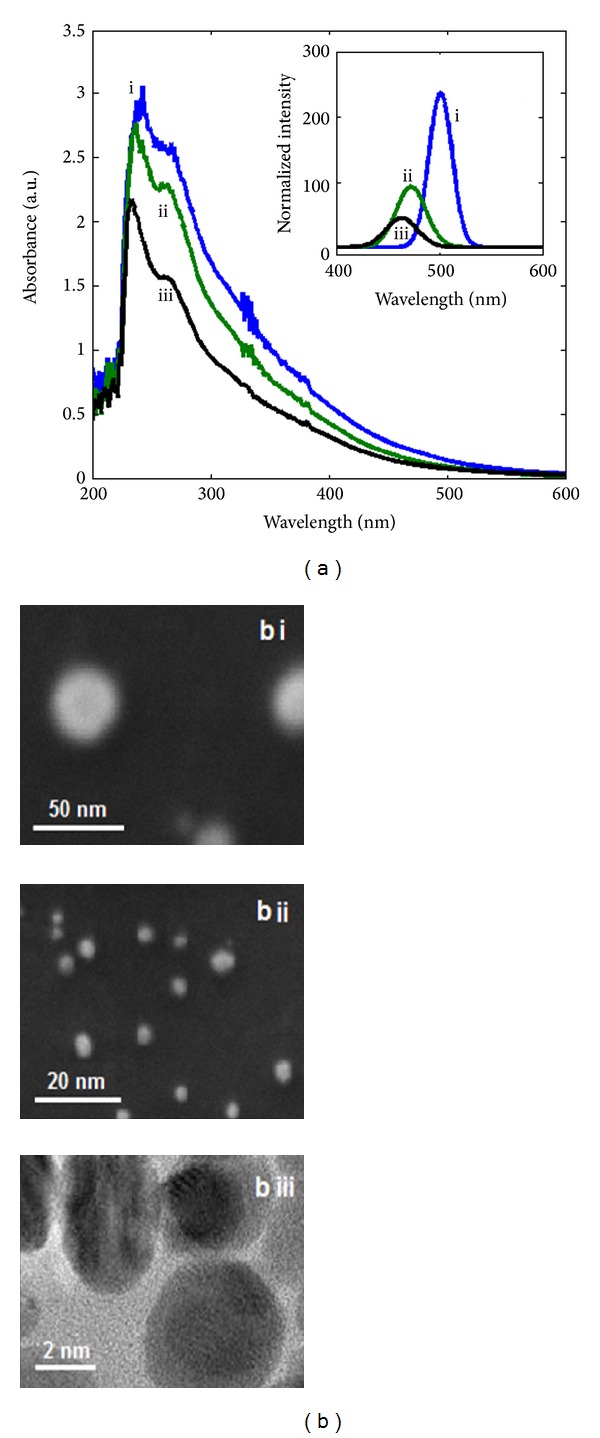
UV-Vis Spectroscopy of separated bands (i, ii, and iii) showing signature absorbance of C-dots. Inset shows PL spectra of corresponding bands (*λ*_ex_ = 250 nm). Right panel displays SEM image (bi and bii) and HRTEM image (biii) of bands i, ii, and iii.

UV-Vis analysis of B1 shows a sharp peak at 243 and a shoulder at 267 nm (Figure 2(a)). Presence of dual peak is signature marker of C-dots as per earlier studies [23]. Origin of intense UV peaks is speculated due to *π* electron transitions in graphene quantum dots (GQDs) containing oxygen as functional groups. Absorbance at 216 nm is due to *π* → π* electron transition of C=C and 273 nm is due to *n* → π* of carbonyl groups [24]. Another notable feature of the spectrum was found to be background absorbance till 600 nm. This may originate due to presence of GO which shows absorbance at higher wavelengths [25]. PL spectra of B1 show a peak at 500 nm (*λ*_ex_ = 250 nm), typical PL emission of carbon nanomaterials including C-dots. PL is unique attribute of quantum confinement as in case of C-dots. Moreover, in case of C-dots including GQDs, excitation dependent emission wavelength (*λ*_em_) is also a signature marker, as elucidated by earlier research [23].

**Figure 3:**
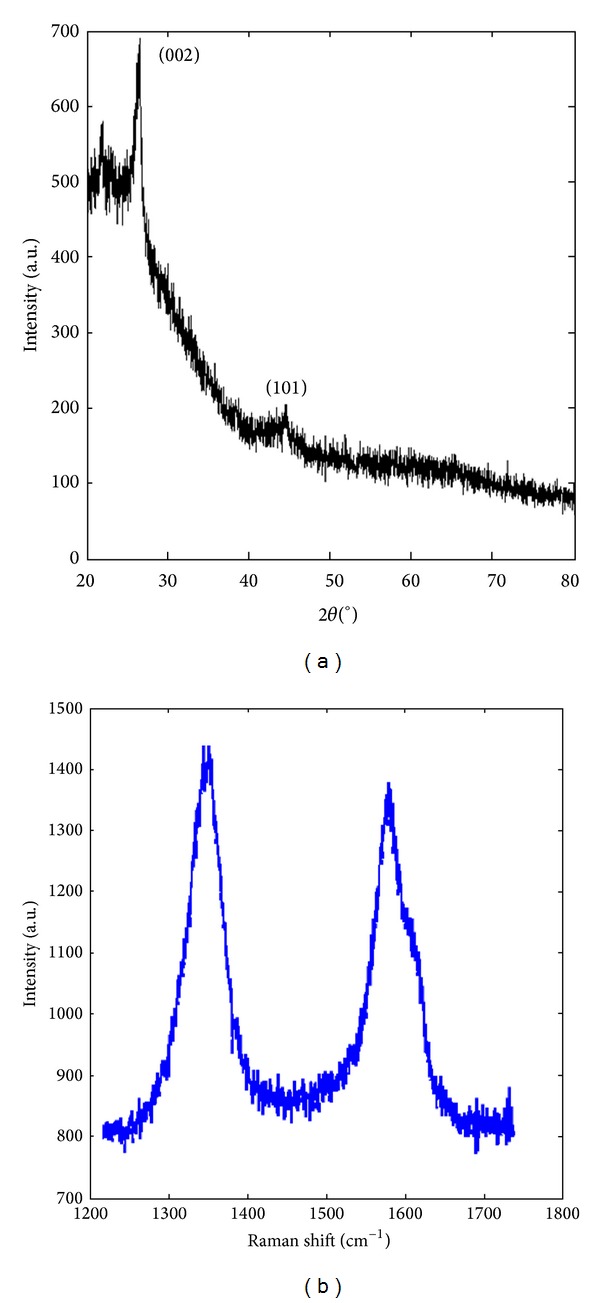
(a) X-ray diffraction pattern and (b) Raman spectra of fraction B1 displaying signature peaks confirming presence of C-dots.

A typical X-Ray diffraction (XRD) (Figure 3(a)) shows prominent peaks at 2*θ* = 25.67° and a feeble peak at 2*θ* = 42.17° which arise due to (002) and (101) diffraction patterns which are of of graphitic carbon, respectively [26] (Figure 2(b)). Raman spectra (Figure 3(b)) of B1 display feeble Raman peak of G-band observed at 1565 cm^−1^ with respect to more intense peaks of D-band at 1303 cm^−1^ showing presence of chaotic carbon nanomaterials in the form of C-dots [27].

Field emission scanning electron microscopy (FE-SEM) image (Figure 2(bi)) shows presence of roughly spherical C-dots of size ~30 nm. In a stark contrast to B1, B2 displayed more prominent green fluorescence blended with blue tinge due to absence of impurity and high concentration of variable sized C-dots. UV-Vis spectra show presence of a sharp peak at 235 nm followed by a hump at 265 nm. In comparison to B1, there was blue shift of 8 nm in UV-Vis spectrum (Figure 2(a)), indicating the reduction in size of C-dots [28]. Moreover, reductions in intensity of the peak as well as the background also suggest relative purity of B2 with respect to B1. PL spectrum (*λ*_ex_ = 250 nm) shows peak at 472 nm, slight blue shift of 8 nm with respect to B1. As per earlier studies PL at lower wavelength indicates C-dots of smaller dimensions [29]. All the other features of B2 such as XRD pattern and Raman spectra were very much similar to B1; hence this data is not shown. SEM image (Figure 2(bii)) shows clear reduction in size to ~10 nm. B3 was light grey color under ambient light and blue under UV light (Figure 1). Blue color displays ultrasmall size [25] as evident from high resolution transmission electron microscopy (HRTEM) image (Figure 2(biii)) showing nanoparticles of size ~7 nm. UV-Vis spectrum (Figure 2(a)) shows deeper UV absorption at 232 nm and a short trail at 263 nm. There was further blue shift of 3 nm due to reduction in size as evident from HRTEM. PL spectrum shows diminished intensity followed by a peak at 463 nm.

**Figure 4:**
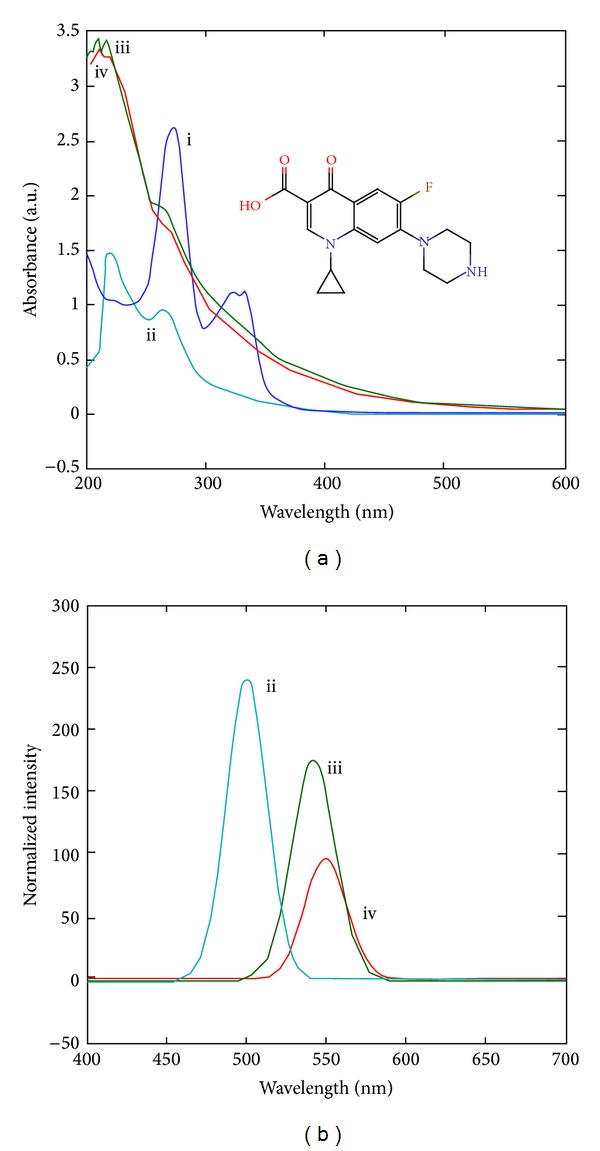
(a) UV-Vis spectra of (i) ciprofloxacin (inset showing its chemical structure), (ii) C-dots, (iii) Cipro@C-dots conjugate, and (iv) postdialysis sample of Cipro@C-dots conjugate and (b) PL spectra of respective samples (ii–iv) with *λ*_ex_ = 250 nm.

In order to further purify C-dots for ferrying ciprofloxacin, B3 was dialyzed against nanopure water for 12 h. Resulting solution was colorless exhibiting bright blue color under UV light (*λ* = 250 nm). This solution was used to fabricate Cipro@C-dots conjugate because of its high stability, small size, and typical PL properties as discussed above. UV-Vis spectrum of ciprofloxacin (Figure 4(a)) shows two distinct peaks at 272 and 330 nm, which arise due to *π* → π* transitions of the fluorobenzene moieties and quinolone ring, respectively [30]. In comparison to dialyzed C-dots (peaks at 218 and 264 nm) and ciprofloxacin, a new peak at 269 nm can be seen which is due to Cipro@C-dots conjugate. Intensity of the peak was found to be deceased after dialysis of the conjugate against nanopure water in order to remove unattached ciprofloxacin (Figure 4(a)). PL intensity of C-dots was also found to be diminished after attachment of ciprofloxacin and a red shift from 442 nm to 540 nm was observed as displayed in Figure 4(b). There can be the following reason for this phenomenon:

- involvement of essential functional groups in formation of chemical interactions which are otherwise responsible for fluorescence of C-dots and/or
- ciprofloxacin induced cross linking of C-dots could also lead to quenching of PL properties.

**Figure 5:**
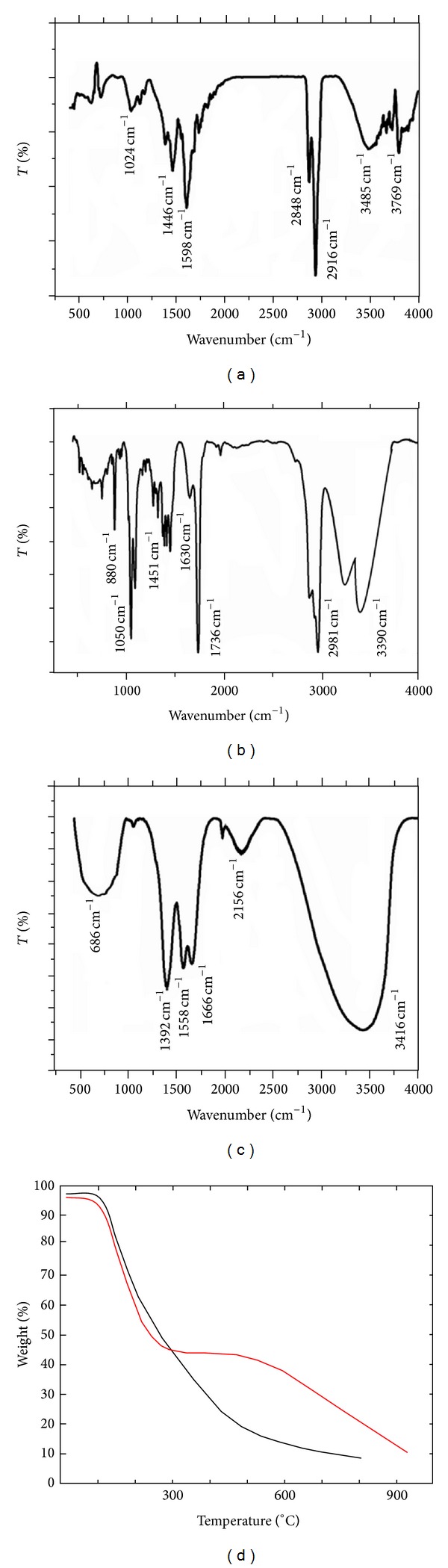
FTIR spectra of (a) bare C-dots, (b) bare ciprofloxacin, (c) Cipro@C-dots conjugate, and (d) TGA of bare C-dots (black) and Cipro@C-dots conjugate (red).

A comparative Fourier transformed infrared (FTIR) spectrum depicting the interaction between C-dots and ciprofloxacin is displayed in Figure 5. Self-passivized C-dots (Figure 5(a)) show typical peaks at 1024, 1446, and 1598 cm^−1^ which can be assigned to C–N stretching, −CH bending, and C=C stretch of aromatic rings, respectively. Other signals correspond to −C-H stretching (both 2848 and 2916 cm^−1^) and alcoholic −OH stretch from aqueous solution and −NH stretch of primary amines (3485 and 3769 cm^−1^) (Figure 5(a)). These are essential functional groups associated with surfaces of C-dots as per previous studies [23].  Figure 5(b) shows bare ciprofloxacin, which, on the other hand, displays typical peaks at 880, 1050, 1451, and 1630 cm^−1^ which arise due to −CH bends of aromatic rings, C–N stretching, −OH bend of carboxylic acid, and C=C stretching, respectively, which are typical molecular signatures of ciprofloxacin [30]. Bands at 1736, 2981, and 3390 cm^−1^ are due to −C=O stretching of carboxylic acids, −CH stretch of alkanes, and −NH stretching of 1° amines, respectively.  Figure 5(c) represents IR spectra of Cipro@C-dots conjugate showing the following differences which confirm their chemical interactions:

- appearance of a new broad peak at 686 cm^−1^ due to new −CH bends of aromatics at metaposition by formation of Cipro@C-dots conjugate;
- shift in the peak from 1446 to 1392 cm^−1^ corresponding to −N-O interaction probably between nitrogen of ciprofloxacin with exposed hydroxyl oxygen of passivized C-dots;
- shift in the peak from 1630 to 1666 cm^−1^ due to new C=C stretching and involvement of amine group (−NH) in the complex. This is further supported by a prominent peak at 1558 cm^−1^ arising due to –NH bends of amides or due to CH_2_ and CH_3_ deformations [31];
- a broad peak at 3416 cm^−1^ was due to −OH stretching of alcohols present on the C-dots as well from the aqueous solution counterpart. There might also be weak interaction present between functionalized C-dots and ciprofloxacin molecules via hydrogen bonding.

From the above findings, chemical interaction involving many weak bonds such as amide linkages and weak hydrogen bonds between carbonyl and amino groups can be speculated.

Comparative thermogravimetric analysis (TGA) displayed in Figure 5(d) shows interaction of C-dots and ciprofloxacin. In case of pure C-dots, weight loss at 108°C can be seen which is due to water molecules associated with C-dots. Consistent loss in weight can be seen which can be speculated due to loss of functional groups associated with C-dots surface. Cipro@C-dots conjugate shows multiple losses in weight. Initial weight loss was the same as earlier case. But, <45% loss in the weight can be seen at 305°C followed by 50% at 585°C. This may be due to blend of strong and weak interaction between C-dots and ciprofloxacin. We could not interpret more from this since there is no report till date of interaction of ciprofloxacin with C-dots.

**Figure 6:**
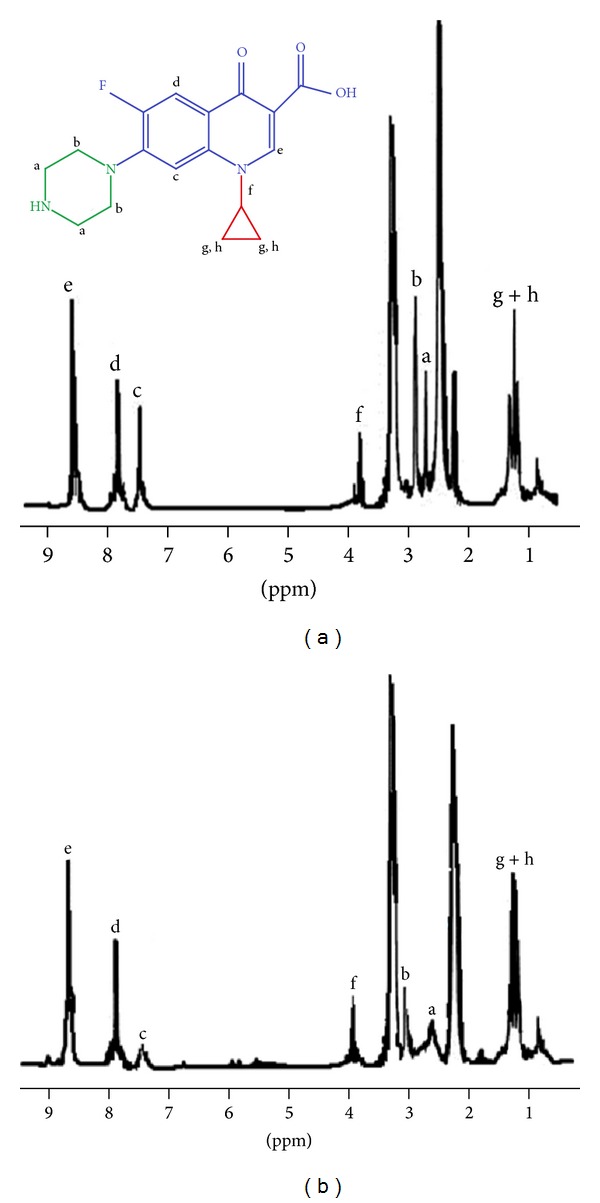
NMR spectra of (a) bare ciprofloxacin and (b) Cipro@C-dots conjugate.

Figure 6 shows NMR spectra of pure ciprofloxacin (Figure 6(a)) and Cipro@C-dots conjugate (Figure 6(b)). Comparative observations of spectrum of ciprofloxacin (inset of Figure 6(a) shows peaks of different structural components of ciprofloxacin) and its conjugate with C-dots reveal the following facts about their interactions:

- ^1^H NMR of pure ciprofloxacin (in DMSO) displays typical peaks at *δ* 0.8, 1.4, 2.3, 2.5, 2.6, 2.8, 3.4, 3.9, 7.4, 7.9, and 8.7;
- in NMR spectra of Cipro@C-dots, there was minor decrease in the intensity of peak at *δ* 7.4 which may be due to weak interaction between −CH of aromatic rings containing fluorine and C-dots surface;
- another peak at *δ* 2.4 (shift from 2.6 to 2.4) in Cipro@C-dots indicates formation of bonds between piperazine moiety of ciprofloxacin and C-dots;
- appearance of the new peak at *δ* 3.3 (from 2.8 to 3.3) also supports the interaction of C-dots with ciprofloxacin involving piperazine moiety.

Release profile of C-dots due to their charismatic surface properties was found to be excellent sink for ciprofloxacin having loading capacity of ~99.8% calculated using (1). During first 3 h, the conjugate showed 3.22 *μ*M ciprofloxacin release which increased to 14.31 *μ*M after 8 h (Table S1). There was a slight increase in release after 12 h (16.41 *μ*M) which became almost steady at ~18 *μ*M even after 48 h. The release mechanism was very much advantageous and followed zero order statistics (SI Scheme 1b). This release behavior is particularly important when passive targeting mechanism is employed. Drug takes time to reach and act on the site of infection; hence it is very important that it must not be metabolized faster on one hand and should not deposit at nonspecific sites. Cipro@C-dots conjugate would provide an advantage of releasing antibiotic at slower rate, whereby giving longer time to reach at the site of infection and facilitate controlled release. This becomes important since nonspecific deposition and use of higher concentration of antibiotics lead to microbial resistance to the drug.

**Figure 7:**
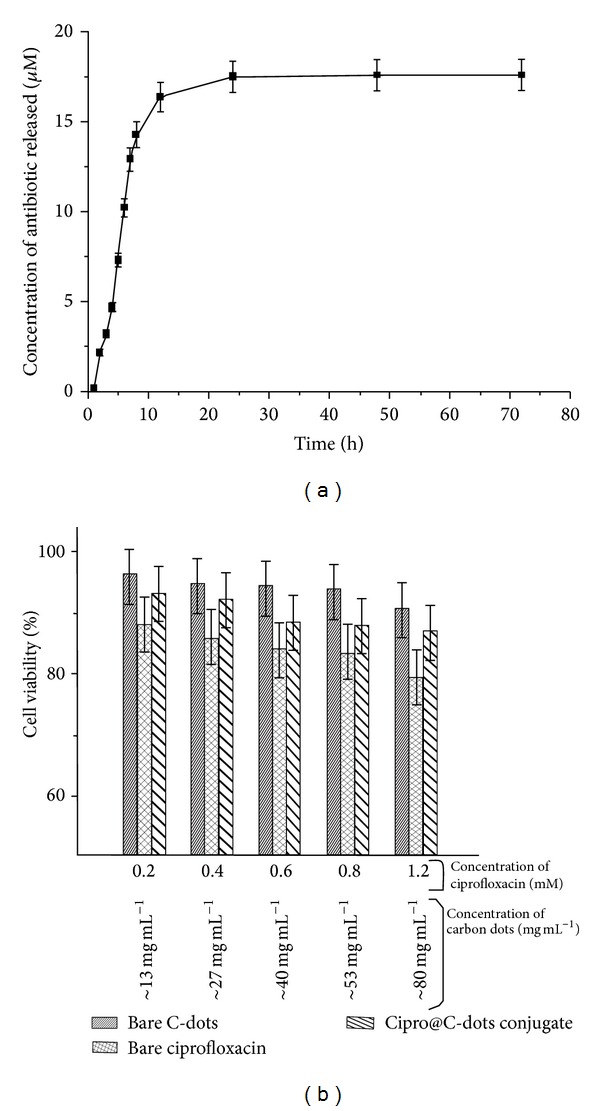
(a) Drug release profile of Cipro@C-dots conjugate under physiological condition (pH 7.4) displaying time-dependent controlled release of ciprofloxacin (error bars represent 5% error) and (b) cytotoxicity of bare C-dots, bare ciprofloxacin, and Cipro@C-dots conjugate on Vero cell lines.

Cytotoxicity studies showed that C-dots were exceptionally biocompatible on Vero cells under ideal conditions of growth (Figure 7(b)). Table S2 summarizes impact of different concentrations on C-dots, free ciprofloxacin, and Cipro@C-dots conjugate on Vero cell lines in terms of percentage viability at various concentrations of test samples. C-dots were found to have negligible impact on Vero cells at all the concentrations (Figure 7(b)). More than 90% cells were found to be healthy after incubation with bare C-dots up to ~80 mg mL^−1^ (Table S2). Free ciprofloxacin was found to be highly inimical than C-dots showing 79% cell viability at its highest concentration (1.2 mM). Cipro@C-dots conjugate was found to be extremely compatible with respect to bare ciprofloxacin. Vero cells showed 93% survival initially which got reduced to 84% at highest concentration having equal concentrations of ciprofloxacin and C-dots as compared to free ciprofloxacin. This may be due to controlled release of antibiotic from C-dots.

**Figure 8:**
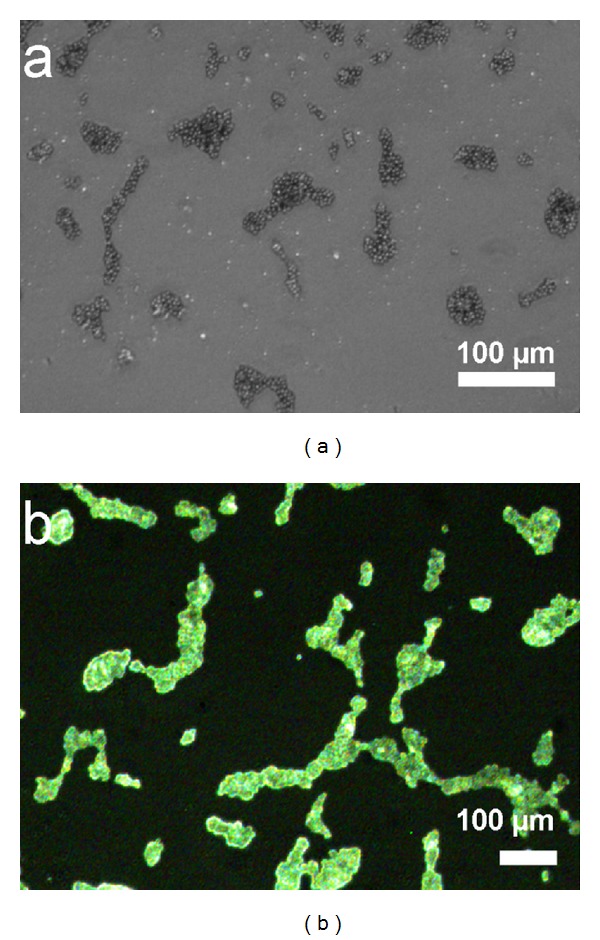
Bioimaging using fluorescent carbon dots.* S. cerevisiae* treated with bare C-dots (13 mg mL^−1^) under (a) normal light and (b) fluorescence (*λ* = 350 nm).

Another significant property of C-dots was realized in microbial imaging as shown in Figure 8. Figures S1 and S2 show green fluorescing bare carbon dots and Cipro@C-dots at their respective concentrations under UV excitation (365 nm), respectively. After incubation for 4 h with yeast (5 × 10^7^ cells mL^−1^), the cells showed bright green fluorescence upon excitation at 350 nm. C-dots were internalized inside the cells (Figure 8(b)) giving excitation dependent green florescence emission. This feature of C-dots can be further used to fabricate molecular tags to view the site of infection when used along with molecular markers on the surface. It would be very interesting to understand the internalization mechanism of C-dots into cells.

Antimicrobial activity of bare C-dots, ciprofloxacin, and Cipro@C-dots was performed on two representative gram positive bacteria,* B. subtilis* and* S. aureus*, and on two representative gram negative bacteria,* E. coli* and* P. aeruginosa*.  Table 1 shows antimicrobial activity results of various samples with their zone of inhibition. Positive control ciprofloxacin being a broad spectrum antibiotic showed distinct zone of inhibition against all bacteria with highest against gram negative* P. aeruginosa* and relatively least against gram positive* B. subtilis*. Bare C-dots on the other hand, as compared to bare ciprofloxacin, showed less antimicrobial activity. The activity might be due to various functional groups present on C-dots which might react with cellular enzymes and inhibit cellular proliferation. In contrast to this, Cipro@C-dots conjugate showed enhanced antimicrobial activity against selective gram strain bacteria. Its activity was highest against gram negative* P. aeruginosa* and relatively less against gram positive* B. subtilis* but more than free C-dots or free ciprofloxacin. It could be inferred here that the antimicrobial activity is retained by the ciprofloxacin and C-dots which are acting in synergism as a potent antimicrobial agent. It must be noted here that the complex also shows slight less activity against* S. aureus* and* E. coli* as compared to bare antibiotic. At the same time, bare C-dots did show potent antimicrobial activity towards these organisms. Hence, it can be hypothesized that may be the antibiotic from the final conjugate was released at a slower rate to act against these organisms. As shown earlier the antibiotic is released in a physiological pH. Hence, “selective synergism” could be the right term to explain this scenario of the antimicrobial potential of Cipro@C-dots conjugate. Nevertheless, this property could be used in simultaneous imaging [32] and drug delivery.

## 4. Conclusions

C-dots can act as efficient nanosink for delivery of therapeutic payloads such as ciprofloxacin due to their excellent biocompatibility, optical properties, and self-passivation properties. Ciprofloxacin can be easily anchored to self-functionalized C-dots without involvement of stringent protocols. Loading capacity of C-dots (>90%) shows it as an ideal vehicle for ferrying significant amount of clinical payloads. Also, path of C-dots can be traced due to its magnificent photoluminescence properties. The conjugate was a potent antimicrobial in nature against both gram positive and gram negative bacteria. Potential antibiotics like ciprofloxacin can be released at sustained rate from the surface of C-dots, following Higuchi model under physiological conditions.

## Supplementary Material

Supplementary material contains quantum yield values, elemental composition, drug loading -release calculations- results, cytotoxicity and fluorescence images.

## Figures and Tables

**Table 1:** Antimicrobial activity of bare C-dots, bare ciprofloxacin, and Cipro@C-dots conjugate on different gram positive and gram negative microorganisms.

Microorganisms	Average diameter of zone of inhibition (mm) ± standard deviation (*σ*)		
	Bare C-dots	Bare ciprofloxacin	Cipro@C-dots conjugate
*B. subtilis *	1.2 ± 0.2	2.5 ± 0.3	3.1 ± 0.2
*S. aureus *	1.4 ± 0.1	2.7 ± 0.2	2.5 ± 0.2
*E. coli *	1.1 ± 0.1	2.6 ± 0.1	2.5 ± 0.1
*P. aeruginosa *	1.3 ± 0.1	3.1 ± 0.1	3.3 ± 0.2
