# Supplementary material for: Antibiotic Conjugated Fluorescent Carbon Dots as a Theranostic Agent for Controlled Drug Release, Bioimaging, and Enhanced Antimicrobial Activity
Source: J Drug Deliv. 2014 Mar 18;2014:282193. doi: 10.1155/2014/282193 (PMC3976943; doi:10.1155/2014/282193)
Supplement: Supplementary file 1 — Supplementary material contains quantum yield values, elemental composition, drug loading -release calculations- results, cytotoxicity and fluorescence images. [file 282193.f1.docx]

**SUPPLEMENTARY INFORMATION**

**Antibiotic Conjugated Fluorescent Carbon dots as a theranostic agent for controlled drug release, bioimaging and enhanced anti-microbial activity**

Mukeshchand Thakur^†^, Sunil Pandey*^†^, Ashmi Mewada^†^, Vaibhav Patil, Monika Khade, Ekta Goshi and Madhuri Sharon

N.S.N. Research Center for Nanotechnology and Bionanotechnology, Ambernath, India. *Corresponding Author: S. Pandey

Email ID: [gurus.spandey@gmail.com](mailto:gurus.spandey@gmail.com)

Phone: (+91) 9004024937

^†^Authors have equal contribution in performing experiments, writing and discussion of the paper.

**S1 Quantum yield calculation and elemental composition**

Quantum yield (QY) of carbon dots purified from separated fractions of SDGC were calculated as was done in our previous report [1]. The quantum yields obtained at following excitation wavelengths- 350, 400 and 450 nm.

For fraction a, QY was obtained as 0.9, 0.8 and 0.7; for fraction b, QY obtained was 0.6, 0.8 and 0.1; and for fraction c, QY was obtained as 0.6, 0.4 and 0.2, when excited at 350, 400 and 450 nm respectively.

Elemental analysis of C-dots used for biological study showed presence of majorly 63.12 % carbon (C), 8.13 % hydrogen (H), 19.82 % oxygen (O) and 8.93 % were lost as loss of ignition (LOI).

**Scheme 1**

1. **Drug loading calculations**-

Initial amount of ciprofloxacin (a) = 1000 μM

Initial amount of drug bound (b) = 998.04 μM

Amount of unbound drug (a-b) = 1.96 μM

Drug loading efficiency = (a-b)/a *100 = 99.8 %

1. **Statistics of drug release**

Mode of Ciprofloxacin release was studied using following equation [2]:

Zero order: Q_t_ = Q_0_ + k_0_t

Where, Qt is the amount of drug released at time t, Q_0_ is the initial amount of drug released, k_0_ is the Zero-order release constant. Table S1 shows the zero order rate kinetics followed by Cipro@C-dots conjugate at physiological pH conditions.

**Table S1 Drug release values with respect to time for calculation of ciprofloxacin release by zero-order kinetics.**

| Time(h) | Antibiotic Release (μM) | Cumulative antibiotic release | Percentage cumulative antibiotic release |
| --- | --- | --- | --- |
| 1 | 0.14 | 0.14 | 0.13 |
| 2 | 2.13 | 2.27 | 2.1 |
| 3 | 3.22 | 5.49 | 5.1 |
| 4 | 4.67 | 10.16 | 9.54 |
| 5 | 7.31 | 17.47 | 16.4 |
| 6 | 10.22 | 27.69 | 26 |
| 7 | 12.92 | 40.61 | 38.13 |
| 8 | 14.31 | 54.92 | 51.57 |
| 12 | 16.41 | 71.33 | 66.98 |
| 24 | 17.533 | 88.863 | 83.45 |
| 48 | 17.622 | 106.48 | 100 |

Note: Antibiotic release is a mean value taken by performing the experiments in triplicate.

**Table S2 Showing cytotoxicity data of bare C-dots, bare ciprofloxacin and Cipro@C-dots conjugate on Vero cell lines. The concentrations represent ciprofloxacin concentrations (mM) either in free state or in the conjugate and corresponding concentration of C-dots (mg ml^-1^) either in free state or in the conjugate.**

|  | Percentage cell viability ± σ | | |
| --- | --- | --- | --- |
| Concentration | Bare C-dots | Bare Ciprofloxacin | [Cipro@C-dots](mailto:Cipro@C-dots) conjugate |
| 0.2 mM/13 mg ml^-1^ | 95.93±0.12 | 87.6±0.4 | 93±0.12 |
| 0.4 mM/27 mg ml^-1^ | 94.16±0.09 | 85.23±0.04 | 91.93±0.12 |
| 0.6 mM/40 mg ml^-1^ | 94.1±0.28 | 83.56±0.33 | 88.13±0.09 |
| 0.8 mM/53 mg ml^-1^ | 93.4±0.40 | 82.73±0.12 | 87.66±0.16 |
| 1.2 mM/80 mg ml^-1^ | 90.23±0.28 | 78.9±0.09 | 84.76±0.24 |

Note: Standard deviation^σ^

An average of data from triplicates were used in calculation of standard deviation from three independent experiments.


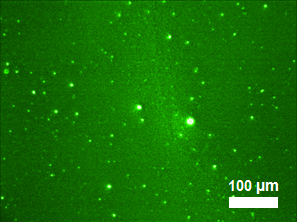


**Figure S1 Green fluorescing bare C-dots at a concentration of 13 mg ml^-1^ under fluorescence microscope upon excitation at 365 nm.**


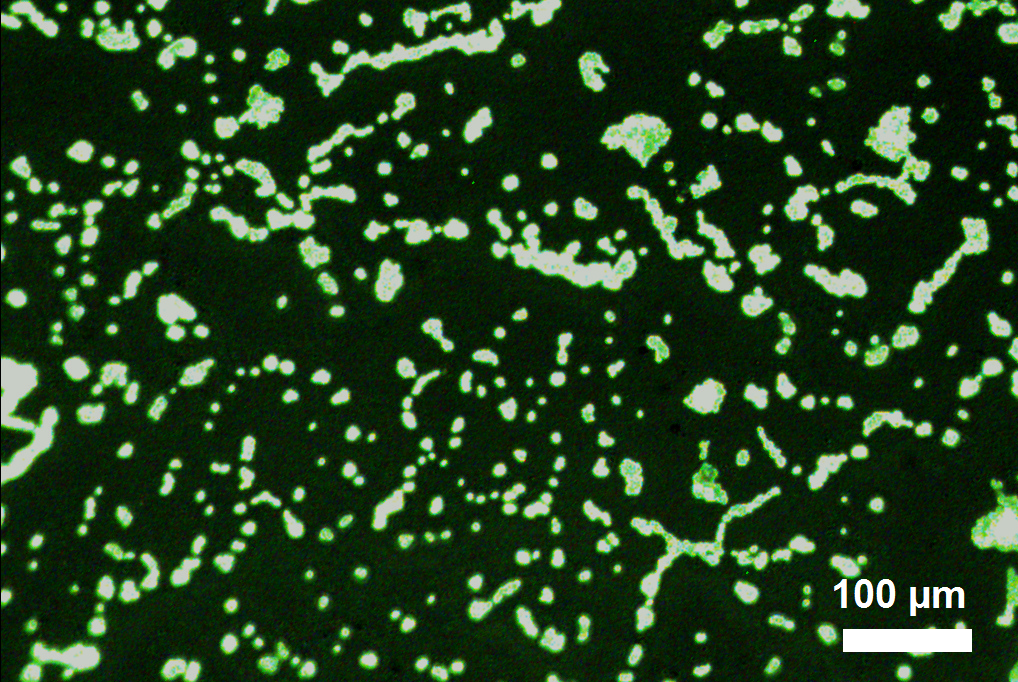


**Figure S2 Showing fluorescence image of *S. cerevisiae* treated Cipro@C-dots at a maximum of 1.2 mM ciprofloxacin (containing a concentration of 80 mg ml^-1^ C-dots) under fluorescence microscope upon excitation at 365 nm.**

**REFERENCES**:

1. Mewada A, Pandey S, Shinde S, Mishra N, Oza G, Thakur M, Sharon M, Sharon M. Green synthesis of biocompatible carbon dots using aqueous extract of Trapa bispinosa peel. Mater Sci Eng C Mater Biol Appl. (2013)1;33:2914-7.
2. Pandey S, Oza G, Mewada A, Shah R, Thakur M, Sharon M. Folic acid mediated synaphic delivery of doxorubicin using biogenic gold nanoparticles anchored to biological linkers. J. Mater. Chem. B, 2013,1, 1361-1370.
